# Study on Reutilization of Pyrolytic Residues of Oily Sludge

**DOI:** 10.1155/2020/8858022

**Published:** 2020-10-23

**Authors:** Chao Tang, Jiaojiao Guan, Shuixiang Xie

**Affiliations:** ^1^State Key Laboratory of Petroleum Pollution Control, Beijing 102206, China; ^2^Chongqing Water Resources and Electric Engineering College, Chongqing 402160, China; ^3^School of Petroleum Engineering, Yangtze University, Wuhan 430000, China

## Abstract

Pyrolytic residues of oily sludge are a kind of hazardous solid waste produced by high-temperature pyrolysis of oily sludge, which still contains a certain amount of mineral oil; improper disposal can cause secondary pollution. In order to reutilize the pyrolytic residues of oily sludge, the pyrolytic carbon in pyrolytic residues is recovered by a combination of physical flotation and chemical separation, and they are used for the treatment of oilfield wastewater and adsorption of oil. The results showed that the purity of the pyrolytic carbon is 95.93%; many pores of different sizes are distributed on the surface, with mainly mesoporous distribution. Specific surface area, pore volume, and average pore diameter reach 454.47 m^2^/g, 0.61 cm^3^/g, and 6.91 nm, respectively. Adsorption effect of pyrolytic carbon on COD and oil in oilfield wastewater is better than that of activated carbon at the same condition. With regard to adsorption on diesel and crude oil, the initial instantaneous adsorption rate of pyrolytic carbon is 3.8 times and 1.86 times faster than that of activated carbon, respectively. When pyrolytic carbon reaches saturated adsorption, cumulative adsorption of activated carbon on diesel and crude oil is much lower than that of pyrolytic carbon.

## 1. Introduction

The requirement of reduction, harmlessness, and reutilization for oily sludge treatment provokes oilfield enterprises and researchers' interest upon the pyrolysis technology, which is regarded as the most promising oily sludge treatment technology owing to its advantages of high reduction effect, high recovery rate of oil and gas resources, and capability to immobilize heavy metals [1–3]. The pyrolytic products of oily sludge include three phases: liquid products are water, low condensation point crude, etc.; gaseous products are usually CH_4_, CO_2_, CO, H_2_, etc.; and solid products are residues in the reactor generally called pyrolytic residues after pyrolysis reaction. At present, research of oily sludge pyrolysis is mainly concentrated on pyrolysis process, output, and properties of pyrolytic oil and gas, and little works have covered on pyrolytic residues with a large portion of pyrolytic products containing unrecovered oil and some heavy metals, which will cause secondary pollution if not disposed properly [4–6]. The pyrolytic residues of oily sludge are listed in “National Catalogue of Hazardous Wastes” in China, and the corresponding disposal has become a bottleneck restricting the development of oily sludge pyrolysis technology in oilfield enterprises [7].

The paper studied on the pyrolytic carbon in pyrolytic residues recovered by the combination of physical flotation and chemical separation, and pyrolytic carbon is used for the treatment of typical pollutants from oilfield wastewater and oil adsorption for the characteristics of its high carbon content in residues after pyrolysis of oily sludge. Therefore, the use of pyrolytic carbon was clarified and the reutilization of pyrolytic residues of oily sludge was realized.

## 2. Experimental

### 2.1. Analytical Method

#### 2.1.1. Composition of Pyrolytic Residues and Their Heavy Metal Pollutants

Pyrolytic residues of oily sludge were from the oily sludge pyrolysis station Liaohe Oilfield. Water content and oil content of residues were measured according to standards GB/T 8929-2006 and SY/T5118-2005, respectively. Residual content was calculated by the method of dispersion. Heavy metal pollutants were analyzed by iCAP RQ ICP-MS.

#### 2.1.2. Characterization of Products

Determination of ash was based on the standard of GB/T7702.15-2008. Carbon purity was calculated by the gravimetric method (the ratio of the product after removing the ash to the mass of the product). The elemental analysis was adopted by Quantax 200XFlash5000-10 EDS. Analysis of surface properties and SEM were performed by using NOVA-2000e N2 adsorption ASAP and Quanta250 tungsten filament scanning electron microscope, respectively. Determination of iodine value was based on the standard of GB/T7702.7-2008.

## 3. Results and Discussion

### 3.1. Composition Analysis of Residues and Their Heavy Metal Pollutants

The composition of pyrolytic residues in Table 1 indicates that the oil content of residues exceeded the control standard of no more than 0.3% mineral oil content regulated in “Pollutant Control Standards for Agricultural Sludge” in China, which cannot be disposed for agricultural use directly.  Table 2 displays the extraction toxicity of heavy metals in pyrolytic residues, which was less than regulation values of “Identification Standards for Hazardous Wastes-Identification for Extraction Toxicity” and the first-order of “Integrated Wastewater Discharge Standard” in China, indicating that the heavy metal pollution will not be caused in the process of reutilization.

### 3.2. Pyrolytic Carbon Recovery from Residues and Its Characterization

#### 3.2.1. Pyrolytic Carbon Recovery Method in Residues

It was identified that the pyrolytic carbon in pyrolytic residues was recovered by a combination of physical flotation and chemical separation after lots of experimental exploration. First, residues were charged into flotation column. Carbon and ash were separated by bubbles under the action of the collector because of the difference between hydrophobicity and hydrophilicity of the surface of carbon and ash. After that, compound acid solution was added to residues at the solid-liquid ratio of 1 : 8, heating reaction was carried out for 2 h, acid-soluble ash was removed by washing, and then compound alkali solution was added at the solid-liquid ratio of 1 : 10, heating reaction was carried out for 2 h, and alkali-soluble ash was removed by washing. After washing and filtering, the filtrate was neutral. And it was dried and the pyrolytic carbon was obtained.

Ash and carbon purity of pyrolytic residues, residues by flotation, acid-soluble product, and pyrolytic carbon were measured, and the results shown in Table 3 indicate that the ash content of pyrolytic residues was as high as 47.43%. After flotation, acid dissolution, and alkali dissolution, the ash content was reduced gradually and the carbon purity was increased steadily. At last, the ash content of pyrolytic carbon dropped to 4.07%, and the purity of pyrolytic carbon reached 95.93%. The element composition of pyrolytic residues, acid-soluble product, and pyrolytic carbon were determined by using the X-ray fluorescence spectrometer. Activated carbon was provided by Hongsheng Activated Carbon Factory and was used for comparative research at the same time. The results shown in Table 4 indicate that the carbon content of the pyrolytic residues was close to 40%, followed by aluminum, silicon, and iron compounds; after flotation and acid treatment, the compounds aluminum, calcium, and iron were removed a lot. After alkali treatment, the compound silicon was removed a lot. After physical flotation and chemical separation of pyrolytic residues, the elemental compositions of the product (pyrolytic carbon) and activated carbon were very similar.

#### 3.2.2. Characterization of Pyrolytic Carbon

**Figure 1:**
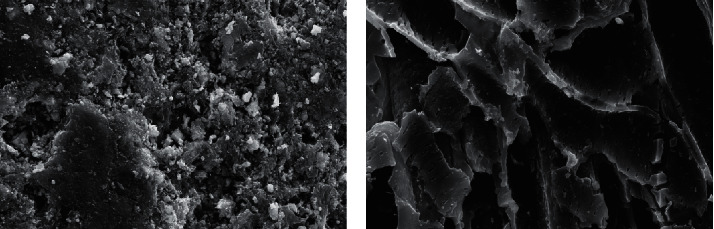
SEM images of (a) pyrolytic carbon and (b) activated carbon.

Surface properties and iodine value of pyrolytic carbon were analyzed, and the comparative study with activated carbon was performed. The results shown in Table 5 indicate that specific surface area of pyrolytic carbon was smaller than that of activated carbon, while pore volume and average pore size were larger; pore size distribution was mainly mesoporous. Pore size of activated carbon was small and was mainly made up of micropores. Iodine value was related to development of micropores [8], which also showed that the development of micropores in activated carbon was better than that of pyrolytic carbon. Surface appearance of pyrolytic carbon and activated carbon was analyzed, respectively, under the scanning electron microscope (SEM). The results shown in Figure 1 display that the surface of pyrolytic carbon was rough, pore distribution was not uniform, and pore size was relatively large, which were consistent with the results of the surface property test. While activated carbon was compact, the pore size was small and distribution was uniform.

### 3.3. Treatment of Oilfield Wastewater by Pyrolytic Carbon

#### 3.3.1. Single-Factor Study

Pyrolytic carbon was used for the treatment of typical pollutants (COD and oil) in oilfield wastewater. Oilfield wastewater was from Liaohe Oilfield, and the wastewater was filtered by the filter paper for removal of oil slick and suspended solids before the experiment. The content of COD in wastewater was measured according to the standard of HJ 924-2017. Determination of oil content was based on the standard of HJ637-2018. COD and oil content in oilfield wastewater was 578.73 mg/L and 52.59 mg/L, respectively. Experimental method: a certain amount of pyrolytic carbon was added into a conical flask filled with 100 mL of oilfield wastewater, placed in a water-bathing constant temperature vibrator for a certain period of time, and then filtered. Then, COD and oil content of the filtrate were determined.

**Figure 2:**
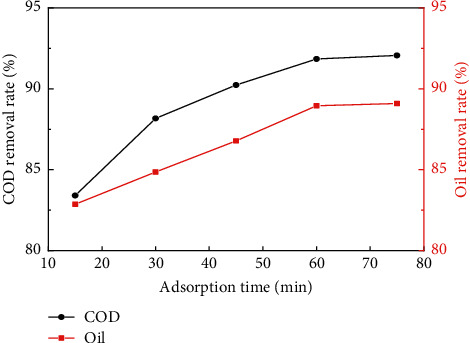
Influence of adsorption time on COD and oil removal rate in oilfield wastewater.

**Figure 3:**
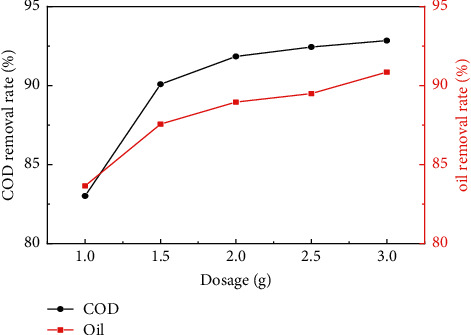
Influence of dosage on COD and oil removal rate in oilfield wastewater.

Figure 2 shows the change of COD and oil removal rate in oilfield wastewater under different adsorption time periods when the dosage of pyrolytic carbon was 2 g, which indicated that with the prolongation of adsorption time, the treatment effect of COD and oil in oilfield wastewater was also increased. When the adsorption time exceeded 60 min, the removal efficiency of COD and oil in oilfield wastewater tended to be stable, while it can be determined that the most suitable adsorption time for pyrolytic carbon to remove COD and oil in oilfield wastewater was 60 min.  Figure 3 shows the changes of COD and oil removal rate in oilfield wastewater under different dosages of pyrolytic carbon when the adsorption time was 60 min, which display that with the dosage of pyrolytic carbon increasing, COD and oil removal rate of oilfield wastewater increased gradually. When the dosage of pyrolytic carbon was 2 g, COD and oil content of oilfield wastewater reduced to 47.22 mg/L and 5.81 mg/L, respectively, which met the requirements for grade 2 of “The National Integrated Wastewater Discharge Standard” in China.

#### 3.3.2. Comparative Study between Pyrolytic Carbon and Activated Carbon

Treatment effect of COD and oil in 100 ml oilfield wastewater was performed with a 2 g adsorbent dosage at 60 min for comparison of pyrolytic carbon and activated carbon. Results shown in Table 6 indicate that the adsorption capacity of pyrolytic carbon was better than that of activated carbon for the treatment of COD and oil in oilfield wastewater. Due to the high proportion of mesopores in pyrolytic carbon, which can provide more effective storage space and diffusion channels for macromolecular organics in oilfield wastewater [9, 10], the microporous structure of activated carbon was not conducive to liquid diffusion and the treatment was not as effective as pyrolytic carbon [11–13].

### 3.4. Adsorption of Oil by Pyrolytic Carbon

**Figure 4:**
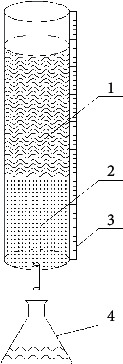
Oil adsorption experiment device. (1) Oil; (2) adsorbent; (3) tick line; (4) collection bottle.

DN 40 mm × 1000 mm plexiglass column was used for adsorption of oil by pyrolytic carbon, diesel, and crude oil which were experimental oils. The device is shown in Figure 4. In the experiment, 200 g of pyrolytic carbon was added into the column, and then 400 mL of oil was injected. Time was counted when the oil was injected, and the downward scale of oil products at regular intervals was recorded. When the oil flowed out of the adsorption column, the time of the first drop of oil flowing out of the column was recorded. This time was the saturated adsorption time of pyrolytic carbon. Through mass-to-volume conversion, oil cumulative adsorption of pyrolytic carbon at different periods of the entire adsorption process was obtained. Then, the instantaneous adsorption rate of pyrolytic carbon at different periods was calculated. Activated carbon was used for a comparative study throughout the experiment.

#### 3.4.1. Study on Instantaneous Adsorption Rate

**Figure 5:**
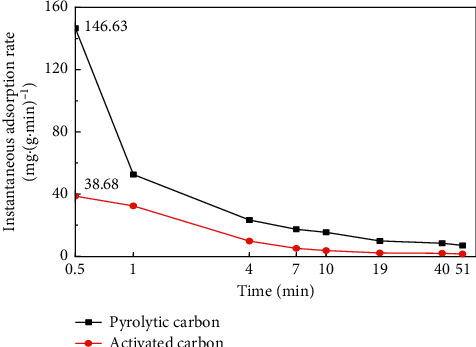
Comparison of instantaneous adsorption rate of pyrolytic carbon and activated carbon on diesel.

**Figure 6:**
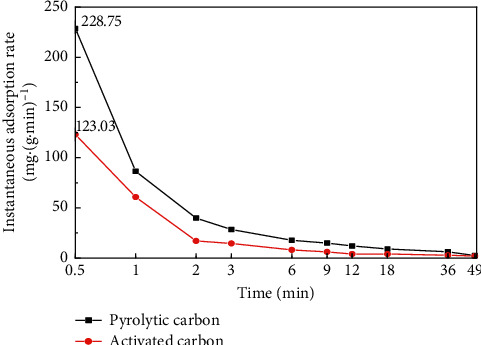
Comparison of instantaneous adsorption rate of pyrolytic carbon and activated carbon on crude oil.

Instantaneous adsorption rate of pyrolytic carbon and activated carbon on diesel was studied, and the results are shown in Figure 5, indicating that, at the initial 0.5 min of the experiment, the instantaneous adsorption rate of pyrolytic carbon on diesel was 146.63 mg/(g·min) and activated carbon was only 38.68 mg/(g·min). The initial instantaneous adsorption rate of pyrolytic carbon on diesel was 3.8 times faster than that of activated carbon. The instantaneous adsorption rate of pyrolytic carbon and activated carbon on crude oil was studied, and the results shown in Figure 6 indicate that the instantaneous adsorption rate of pyrolytic carbon on crude oil was 228.75 mg/(g·min) and activated carbon was 123.03 mg/(g·min) at the initial 0.5 min; the initial instantaneous adsorption rate of pyrolytic carbon on crude oil was 1.86 times faster than that of activated carbon. When the adsorption time was extended, the instantaneous adsorption rate of pyrolytic carbon and activated carbon was reduced both on diesel and crude oil. However, the instantaneous adsorption rate of pyrolytic carbon was always faster than that of activated carbon. It can be seen that if an oil spill accident was encountered, pyrolytic carbon can prevent the spilled oil from spreading and polluting the environment quickly because of its rapid adsorption performance, and the advantage was more obvious than that of activated carbon.

#### 3.4.2. Study on Cumulative Adsorption

**Figure 7:**
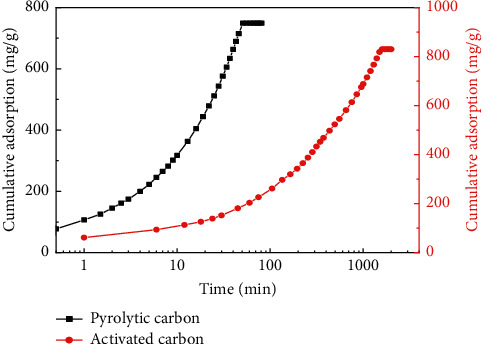
Cumulative adsorption comparison of pyrolytic carbon and activated carbon on diesel.

**Figure 8:**
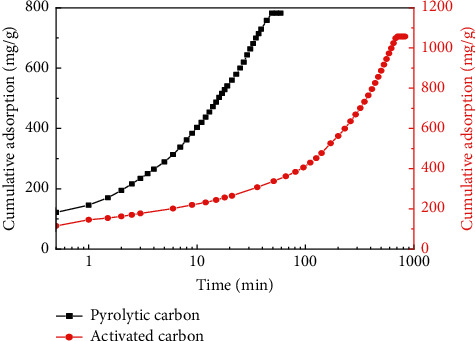
Comparison of cumulative adsorption of pyrolytic carbon and activated carbon on crude oil.

Cumulative adsorption of pyrolytic carbon and activated carbon on diesel and crude oil was studied, and the results are shown in Figures 7 and 8, indicating that the adsorption of pyrolytic carbon on both diesel and crude oil reached saturated adsorption in 60 min, and saturated adsorption capacities were 748.69 mg/g and 787 mg/g, respectively. Saturated adsorption capacity of activated carbon was larger than that of pyrolytic carbon, but saturated adsorption time was longer; adsorption time of crude oil to reach saturated adsorption time exceeded 700 min, and adsorption on diesel was more than 1600 min. When pyrolytic carbon reached adsorption saturation, cumulative adsorption of activated carbon on diesel and crude oil was much lower than that of pyrolytic carbon.

## 4. Conclusions

The pyrolytic carbon in pyrolytic residues of oily sludge was recovered by a combination of physical flotation and chemical separation. The content of carbon in pyrolytic carbon reached 91.03%, and the elemental composition was similar to that of activated carbon.

The surface of pyrolytic carbon was rough and pores of various sizes were distributed, and the pore size distribution was mainly mesoporous. Specific surface area, pore volume, average pore size, and iodine value reached 454.47 m^2^/g, 0.61 cm^3^/g, 6.91 nm, and 327.71 mg/g, respectively.

When the dosage was 2 g and adsorption time was 60 min, the treatment effect of pyrolytic carbon on COD and oil in oilfield wastewater was better than that of activated carbon. The treated oilfield wastewater can reach the requirements for grade 2 of “The National Integrated Wastewater Discharge Standard” in China. With regard to adsorption on diesel and crude oil, the initial instantaneous adsorption rate of pyrolytic carbon was 3.8 times and 1.86 times faster than that of activated carbon, respectively. Saturated adsorption capacity of activated carbon was larger than that of pyrolytic carbon, but it takes a long time to reach saturated adsorption. When pyrolytic carbon reached adsorption saturation, cumulative adsorption of activated carbon on diesel and crude oil was much lower than that to pyrolytic carbon.

## Figures and Tables

**Table 1:** Contents of pyrolytic residues.

Sample	Water content, *w* (%)	Oil content, *w* (%)	Residue content, *w* (%)
Pyrolytic residues	7.81	1.83	90.36

**Table 2:** Extraction toxicity of heavy metals in pyrolytic residues (mg/L).

Sample	Cr	Hg	Ni	Cu	Zn	Cd	Pb	As
Pyrolytic residues	0.036	0.002	0.031	0.046	0.033	0.001	0.024	0.016
A	15	0.1	3	100	100	1	5	5
B	1.5	0.05	1	0.5	2.0	0.1	1.0	0.5

A: regulation values of “Identification Standards for Hazardous Wastes-Identification for Extraction Toxicity”; B: first-order of “Integrated Wastewater Discharge Standard.”

**Table 3:** Ash and carbon purity of pyrolytic residues at different stages.

Sample	Ash, *w* (%)	Carbon purity, *w* (%)
Pyrolytic residues	47.43	52.57
Physical flotation product	40.97	59.03
Acid-soluble product	29.7	70.3
Pyrolytic carbon	4.07	95.93

**Table 4:** Elemental analysis of pyrolytic residues at different stages.

Sample	Element type and mass fraction (%)							
	C	O	Na	Al	Si	S	Ca	Fe
Pyrolytic residues	38.77	21.12	1.16	21.31	12.14	0.94	1.88	2.67
Acid-soluble product	58.48	18.81	—	1.36	20.67	0.68	—	—
Pyrolytic carbon	90.75	6.42	—	1.87	0.66	0.30	—	—
Activated carbon	91.03	7.18	—	0.85	0.33	0.61	—	—

**Table 5:** Surface characteristics and iodine value of pyrolytic carbon and activated carbon.

Sample	Specific surface area, m^2^/g	Pore volume, cm^3^/g	Average pore size, nm	Iodine value, mg/g
Pyrolytic carbon	454.47	0.61	6.91	327.71
Activated carbon	964.34	0.51	2.07	776.3

**Table 6:** Removal capacity comparison between pyrolytic carbon and activated carbon.

Sample	COD content after adsorption (mg/L)	Oil content after adsorption (mg/L)	COD removal rate (%)	Oil removal rate (%)
Pyrolytic carbon	47.22	5.81	91.84	88.95
Activated carbon	87.63	9.45	84.86	82.03

## Data Availability

The table data, figure data, and other related data used to support the findings of this study are included within the article.
